# Dual-edged mechanisms of α-tomatine in hepatocellular carcinoma by suppression of Wnt/β-catenin signaling versus RelB-Driven resistance in tumor therapy

**DOI:** 10.3389/fphar.2025.1616975

**Published:** 2025-09-22

**Authors:** Tianzhen Wang, Chenyue Xu, Yanling Yuan, Bianhong Zhang

**Affiliations:** Shanghai Key Laboratory of Regulatory Biology, Institute of Biomedical Sciences, School of Life Sciences, East China Normal University, Shanghai, China

**Keywords:** α-tomatine, hepatocellular carcinoma, wnt/β-catenin signaling, RelB, Chemoresistance

## Abstract

**Background:**

The plant-derived steroidal alkaloid α-tomatine has emerged as a promising pan-cancer therapeutic agent, its multifaceted biological effects in HCC remain unexplored. This study aims to decipher α-tomatine’s molecular duality in HCC, resolving its paradoxical capacity to simultaneously activate tumor-suppressive signaling and provoke chemoresistance networks, ultimately establishing synergistic phytotherapy strategies.

**Methods:**

HepG2 and SMMC-7721 hepatocellular carcinoma cells were exposed to α-tomatine to evaluate dose-dependent effects on proliferation, migration/invasion, and cell cycle distribution. Transcriptomic profiling via RNA sequencing identified dysregulated pathways. Pharmacological interventions using Wnt3a (activation) and XAV939 (inhibition) modulated Wnt/β-catenin signaling, while CRISPR/Cas9-mediated *RelB* knockout and plasmid-based overexpression established isogenic cell models. These interventions were subsequently applied in BALB/c nude mouse xenografts, where tumor volume was longitudinally monitored during α-tomatine treatment.

**Results:**

α-Tomatine demonstrated dose-dependent suppression of hepatocellular carcinoma cell proliferation, migration, and invasion, concomitant with G2/M phase arrest. Mechanistically, it exerted Wnt/β-catenin inhibition via β-catenin phosphorylation/degradation while paradoxically inducing RelB-mediated reduction of anti-tumor activity. Wnt activation attenuated therapeutic effects, whereas Wnt inhibitors enhanced efficacy. Genetic RelB ablation potentiated α-tomatine’s anti-tumor activity, contrasting with resistance in RelB-overexpressing models. Xenografts confirmed enhanced suppression in RelB-deficient tumors.

**Conclusion:**

This plant-derived alkaloid exerts anti-HCC effects through Wnt pathway modulation, while compensatory RelB activation constrains therapeutic outcomes. Strategic RelB co-targeting establishes a dual pathway phytotherapy paradigm, synergistically merging botanical pharmacodynamics with precision oncology.

## Introduction

Liver cancer is one of the top five common causes of cancer-related deaths worldwide, and it is the only one among these five most fatal cancers whose incidence has been increasing year by year (Siegel et al., 2019). The overall 5-year survival rate for patients with advanced liver cancer is less than 5%. In developing countries, the incidence of liver cancer is even higher (Starley et al., 2010). Primary liver cancer is mainly composed of 90% hepatocellular carcinoma (HCC) and 10% cholangiocarcinoma (CCA). The major risk factors for liver cancer include hepatitis B virus, hepatitis C virus, liver cirrhosis, non-alcoholic fatty liver disease, smoking, obesity, diabetes, iron overload, various dietary exposures, and certain genetic factors (Center and Jemal, 2011). The prognosis of liver cancer is extremely poor, with only 5%–15% of patients eligible for surgical resection, and this only applies to those in the early stages. Treatment options for advanced stages include transarterial chemoembolization (TACE), which can improve the 2-year survival rate by 23% when compared to conservative treatments for patients with intermediate-stage HCC; or oral sorafenib, which is the primary treatment option for advanced cases. However, less than one-third of patients benefit from these treatments, and drug resistance significantly increases within 6 months of starting the treatment regimen (El-Serag et al., 2008). Therefore, further research is needed to find better treatment methods for liver cancer.

α-tomatine is the major saponin in tomato (Lycopersicon esculentum). It provides effect of antimicrobial, antifungal, immunopotentiating activities and anti-inflammatory (Chiu and Lin, 2008; Friedman, 2002; Morrow et al., 2004; Simons et al., 2006). It was reported to promote anti-proliferative effects against human colon HT-29, liver HepG2, breast MCF-7 and stomach AGS cancer cells (Friedman et al., 2009). Moreover, *in vivo* experiments demonstrated α-tomatine shows great antitumor potential in mice mammary adenocarcinoma (Tomsik et al., 2013). The α-tomatine exerts its anti-tumor effects by inhibiting the phosphoinositide 3-kinase/protein kinase B (PI3K/Akt) and extracellular signal-regulated kinase (ERK) signaling pathways in lung adenocarcinoma A549 cells, induces apoptosis in human prostatic adenocarcinoma PC-3 cells by inhibition of NF-κB (Lee et al., 2011) and in liver cancer HepG2 cells by inducing p53, Ca^+2^, and ROS signaling (Echeverria et al., 2022). The aforementioned information has shown that the natural compound α-tomatine is highly effective in inhibiting the growth of multiple cancer cells.

The WNT signaling pathway is a central hub that coordinates multiple cellular signaling cascades. It plays an indispensable role in a wide range of physiological processes, including proliferation, differentiation, apoptosis, migration, invasion, and tissue homeostasis (Liu et al., 2022). The Wnt/β-catenin signaling pathway has been shown to exert indispensable functions on the tumorigenesis of different gastrointestinal cancers, including HCC (Chen et al., 2018; Thompson and Monga, 2007; White et al., 2012). Overexpression of sFRP3, a negative regulator of Wnt pathway, were found to provide an increase of Bax and promote apoptosis in HCC cells (Fang et al., 2021). Suppression of β-catenin resulted in increased expression of apoptosis related genes and an induction of tumor cell apoptosis in liver cancer cells (Fang et al., 2021).

The NF-κB transcription factor family is composed of five members in mammals: RelA (p65), cRel (Rel), RelB, NF-κB1 (p50 and the precursor p105) and NF-κB2 (p52 and the precursor p100) (Oeckinghaus and Ghosh, 2009). RelB is the major subunit of the alternative NF-κB signaling pathway, triggering effective transcription activation after forming the dimer with p52 (Baud and Collares, 2016). Numerous literature using the RelB^−/−^ mice have shown that RelB plays important roles in biological processes including lymphoid organogenesis, B cell maturation, T-cell homeostasis, dendritic cell maturation, autoimmunity and immune response. The deficiency of RelB cannot be compensated by other NF-κB subunits functionally (Grinberg-Bleyer et al., 2018; Guo et al., 2008; Weih et al., 1995). Previous studies have demonstrated the roles of the alternative NF-κB activity, as well as RelB, in the tumorigenesis. The expression level of RelB correlates with prostate cancer patient’s Gleason score (Lessard et al., 2005), and RelB plays a role in resistance of radiotherapy in aggressive prostate cancer (Wei et al., 2017). Upregulated transcriptional activation of RelB by enhancer of zeste homology 2 (EZH2) drives self-renewal and tumor-initiating cell phenotype of triple-negative breast cancer cells (Lawrence and Baldwin, 2016). RelB is not only a prognostic marker for non-small cell lung cancer (NSCLC) patients, but also promotes migration and invasion, and exerts its radio-protective roles in the NSCLC cells (Qin et al., 2018; Qin et al., 2016).

Current evidence suggests that both the Wnt/β-catenin signaling pathway and RelB play crucial roles in regulating tumor progression and modulating therapeutic responses in various solid malignancies. While α-tomatine has demonstrated broad-spectrum anti-tumor activity across multiple cancer types, its precise molecular mechanism in hepatocellular carcinoma remains incompletely understood. Through RNA sequencing analysis, our study reveals that α-tomatine exerts its anti-tumor effects in HepG2 cells partially through suppression of the Wnt/β-catenin signaling axis. Notably, we identified a paradoxical upregulation of RelB expression following α-tomatine treatment, which mechanistically correlated with acquired resistance to the compound’s growth-inhibitory effects. These findings provide the first experimental evidence that: α-tomatine can effectively target Wnt-mediated oncogenic pathways, and RelB serves as a compensatory oncoprotein mediating therapeutic resistance in α-tomatine-treated liver cancer cells.

## Materials and methods

### Cell preparation

HepG2 cells were cultured in DMEM, supplemented with 10% FBS, penicillin (100U/mL) and streptomycin (100 μg/mg). Cells were incubated at 37°C in a humidified atmosphere of 95% air and 5% CO_2_.

### Phytochemicals and reagents

α-tomatine (purity>97%), XAV939 and RS47 were purchased from Selleck (Shanghai, China). Wnt3a was purchased from Peprotech (Shanghai, China). Dimethyl sulfoxide (DMSO) was purchased from Sigma Aldrich (Shanghai, China). Penicillin/streptomycin, DMEM, RPMI-1640, fetal bovine serum (FBS), Lipofectamine 3,000 transfection reagent and MTT (3-(4,5-dimethylthiazol-2-yl)-2,5-diphenytetrazodium bromide) were purchased from Invitrogen (Shanghai, China).

### Wound-healing assay

The cells were seeded at a density of 6 × 10^5^ cells per well on 12-well plate and grown to about 90% confluence before assay. After removing the medium, the cells were wounded by manually scraping with plastic tip. Then the cells were pre-treated with α-tomatine (2 μM) or DMSO for 12 h before scaraping. The images were captured every 12 h post wounding with microscope.

### *In vitro* transwell invasion assay

Cells were treated with α-tomatine (2 μM) or DMSO for 12 h before resuspending. After treatment with α-tomatine or DMSO, the cells were plated at 100 μL cell suspension in chambers with pores (Corning). These chambers were then put in wells with 10% FBS fresh medium. After incubation at 37°C in 5% CO2 for 24°h, the cells invaded the surface of filter were fixed by 4% paraformaldehyde and stained with crystal violet. The images were captured by microscope.

### Propidium iodide (PI) staining for cell cycle assay

Cell cycle was examined by the staining method using PI (BD bioscience, Shanghai, China) according to the manufacturer’s instructions. Briefly, control (DMSO) and α-tomatine (2 μM) treated cells treated cells were collected after 12 h of each treatment, washed in cold PBS twice before fixation with 70% ethanol for 15 min and stained with PI dyes for 15 min under room temperature. Then we used Flow Cytometry 500 to detect the PI stained cells.

### Western blot analysis

Proteins were extracted with RIPA buffer (10 mM Tris, 150 mM NaCl, 0.5% NP-40, 0.1% SDS, 0.1% deoxycholate, 1 mM PMSF, 2 mM sodium fluoride, and 1 mM sodium orthovanadate), then centrifuged at 12,000 rpm for 30 min at 4°C. Briefly, equivalent amounts of proteins were analyzed by 12% SDS-PAGE, then transferred to PVDF membranes (Millipore, Shanghai, China), which were then incubated with specific primary antibodies anti-β-actin (1:5,000, Cat a1978, Sigma), anti-tubulin (1:5000, Cat T8203, Sigma), anti-RelB (1:1000, Cat 4922, CST), anti-p100 (1:1000, Cat 05–361, Sigma), anti-IKK1 (1:1000, Cat 2682, CST), β-catenin (1:2000, Cat 9582, CST), anti-Axin2 (1:1000, Cat 20540-1-AP, proteintech), anti-cyclin A (1:1000, Cat sc-271682, Santa Cruz), anti-cyclin B1 (1:1000, Cat MA1-155, ThermoFisher), anti-p27 (1:1000, Cat sc-1641, Santa Cruz). Finally, proteins were visualized with peroxidase-coupled secondary antibody, using the ECL system for detection.

### RNA-seq and bioinformatic analysis

RNA was used for RNA-seq according to the manual of Illumina bead arrays. Briefly, libraries were prepared by using 1 μg of RNA per samples measured with Qubit 2.0 fluorometer (Thermo Fisher Scientific) by the KAPA stranded mRNA-seq Kit Illumina platform KR0960. Final libraries were tested via agarose gel and multiplexed with a maximum of 24 samples per sequencing reaction. Libraries were sequenced using an Illumina HiSeq 3,000 with single-end 50 bp reads.

Reads were trimmed using cutadapt (cutoff q = 20) and mapped to the mm10 genome. Processed reads showed high quality reads and alignment scores. Count per minute (cpm) values were generated using edgeR to normalize the raw counts data based on sequencing depth. Induced genes were selected by using a cutoff of fold change greater than 1.5 for any treatment time point relative to the control. Transcripts with empty gene names were removed. Data were z-scored and plotted using the heatmap R package. Gene ontology and IPA analysis were performed using Enrichr and Qiagen Ingenuity Pathway Analysis. Anti-tumor drug treated cancer related dataset, GSE235401, GSE272162, and GSE164561 were selected from GEO database (http://www.ncbi.nlm.nih.gov/geo), and the data characteristics were assessed using the GEO2R tool, which generated volcano diagrams.

### CRISPR/Cas9 knocking out of RelB and plasmid

The gRNA sequence targeting RelB 5′-TCG​CCG​CGT​CGC​CAG​ACC​GC-3′ was designed using CRISPOR (www.crispor.tefor.net). The gRNA was subcloned into the LentiCRISPR Puro v2 (Cat 98290, Addgene, China). HepG2 cells were pre-cultured to 60%–80% confluence in 6-well plate and transfected using Lipofectamine 2000 (Cat 12566014, Thermofisher, China) for 6 h. To obtain stably transfected clones, cells were selected in the medium containing Puromycin (10 μg/mL, Cat ant-pr-1, Invivogen, China) for 7 days. The cDNA sequence of human full length RelB was subcloned into pBabe plasmid (Cat 17383, Addgene, China) for overexrepsssion of RelB.

### Luciferase assay

To measure Wnt/β-catenin signaling activity, the 0.3 μg of Super8XTOPFlash and 0.01 μg of Renilla luciferase plasmids were transfected in HepG2 cells followed by Wnt3a (250 ng/mL) and α-tomatine (2 μM) treatment for 12 h or in HepG2 cells with overexpression of RelB or RelB knockout, and the luciferase activities were measured. To measure NFkB signaling activity, the 0.2 μg of 5XNFkB reporter and 0.01 μg of Renilla luciferase plasmids were transfected in HepG2 cells followed by α-tomatine (2 μM) or Wnt3a (250 ng/mL) treatment for 12 h, and the luciferase activities were measured.

### Cell viability and proliferation assay

MTT assay was used to measure cell viability according to manufacturer’s instruction. Cells were plated at a density of 4 × 10^4^/well into 96-well plates and treated with indicated regents. Before measurement, 10 μL MTT solution was added to each well and cells were incubated at 37°C for 4–6 h. Formazan crystals dissolved in 150 μL DMSO were added for 10 min with agitation. Absorbance was measured at 490 nm in a plate reader with SPECTROstarNano (BMG Labtech).

### Mouse HepG2 xenograft model

Six-week old female BALB/c nude mice were obtained from the Jackson Laboratory. 5 × 10^6^ HepG2 cells in PBS were implanted subcutaneously into the right flank of nude mice. The mice were randomly divided into three groups and treated with vehicle (corn oil, Cat C8267, Sigma), α-tomatine (25 mg/kg) or α-tomatine (25 mg/kg) together with Wnt3a (2 ug/kg) everyday by i.p. injection. For the injection of WT and RelB KO HepG2 cells, 5 × 10^6^ WT or RelB KO HepG2 cells in PBS were implanted subcutaneously into the right flank of nude mice. After injection of WT or RelB KO HepG2 cells, the mice receiving each cells were randomly divided into two groups and treated with vehicle or α-tomatine (25 mg/kg) everyday by i.p. injection (n = 5). The mice were sacrifieced 21 days later after injection, and the tumors were weighed and photographed. All the animals were treated according to protocols approved by Animal Care and Use Committee of the East China Normal University and ARRIVE guidlines.

### Statistical analysis

Prism software (GraphPad Software) was used for statistical analyses. Values are shown as mean ± s.e.m. Statistical significance between two samples was determined with two-tailed Student’s t-test. Statistical differences of time courses were assessed by a one-way ANOVA (Kruskal-Wallic) followed by Dunn’s *post hoc* test. *P < 0.05; **P < 0.01; ***P < 0.001.

## Results

### A-tomatine suppresses hepatocellular carcinoma proliferation and migration through G2/M phase cell cycle arrest

**FIGURE 1:**
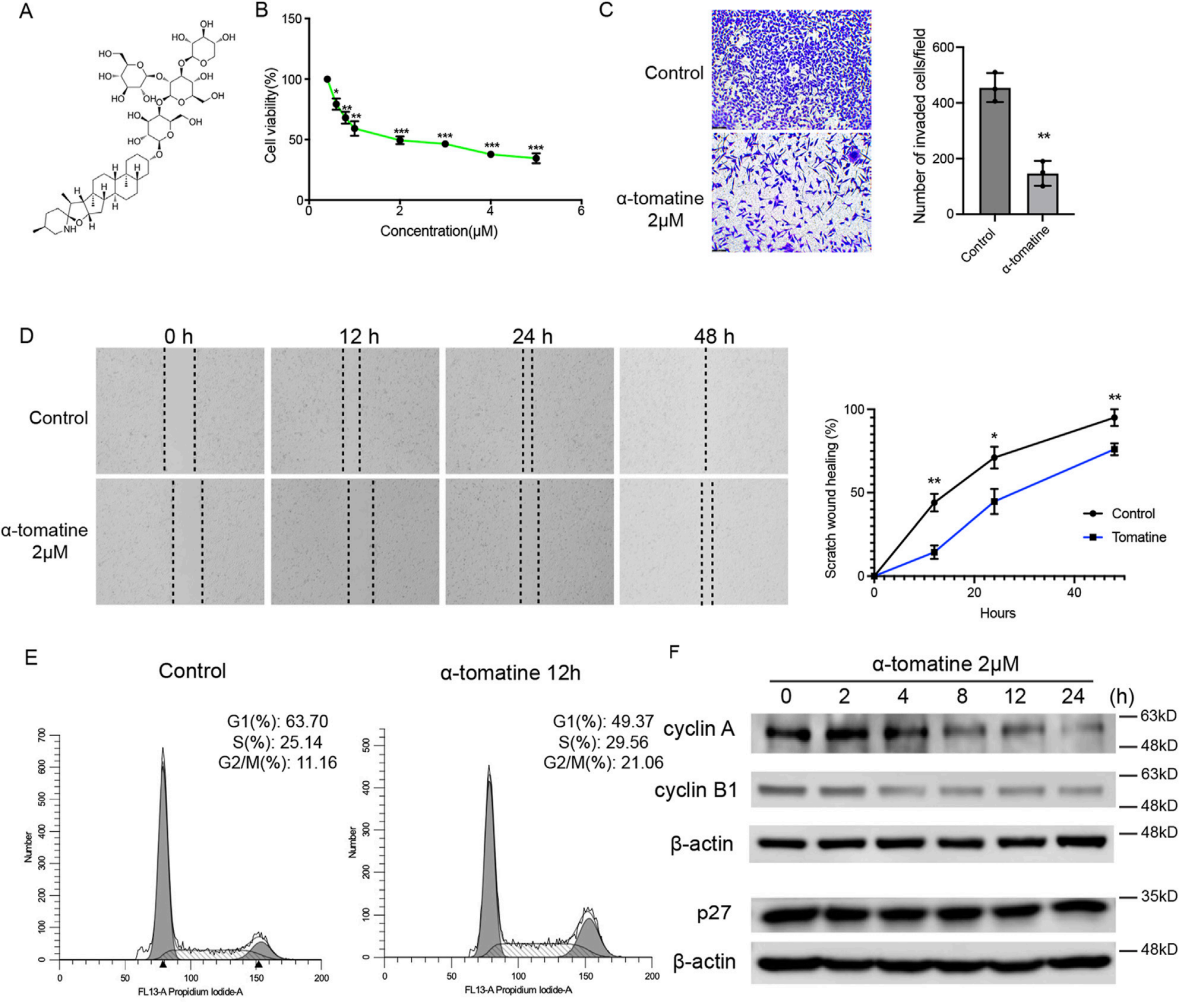
The effect of α-tomatine on cell viability, migration, invasion and cell cycle arrest in liver cancer cell. **(A)** The chemical structure of a-tomatine. **(B)** Cells were treated with a-tomatine for 24 h and cell proliferation was measured using MTT assay. Three biological replacates in each group. **(C)** The transwell invasion assay was performed after 12 h treatment of 2 μM α-tomatine. Number of invaded cells per field was shown. Three biological replacates in each group. **(D)** HepG2 cells were pretreated with DMSO or 2 μM α-tomatine for 12 h, followed by the wound healing assay. Percentage of scratch wound healing was shown. Three biological replacates in each group. **(E)** Assessment of cell cycle using flow cytometry with staining of PI after exposure to 2 μM α-tomatine for 12 h in HepG2 cells. Three biological replacates in each group. **(F)** The HepG2 cells were treated with 2 μM α-tomatine for different time points, and the protein levels of cell cycle regulatory genes were measured by Western blot analysis. All the values are shown as mean ± SEM. Statistical significance was shown as *P < 0.05; **P < 0.01; ***P < 0.001.

α-Tomatine is a type of tomatine alkaloid extracted from tomatoes, composed of a sugar group (tomatine) and a tetrasaccharide group (β-tetra-glucose). The tetrasaccharide group consists of two glucose molecules, one galactose, and one xylose; the four monosaccharides form a branched structure attached to the C-3 position of the sugar (Figure 1A). To evaluate the effect of α-tomatine on cell viability of liver cancer cells, MTT assay was performed. Treatment of α-tomatine to HepG2 cells resulted in a significant dose-dependent (from 0.2 to 5.0 μM) inhibition of cell growth (Figure 1B). The EC_50_ value at 24 h post-treatment with α-tomatine for HepG2 cells was estimated at 0.5418 ± 0.077 μM. Results of HepG2 cells treated with α-tomatine were used for wound healing assays (Figure 1C) and transwell migration assays (Figure 1D) showed that α-tomatine significantly inhibited the ability of cell migration of HepG2 cells. Cell cycle distribution of the treated cells was assessed by labelling the cell’s DNA with Propidium (PI). The G2/M population after α-tomatine treatment were markedly increased compared to control cells after α-tomatine treatment for 12 h (Figure 1E). To further investigate the mechanism of α-tomatine induced cell cycle arrest in HepG2 cells, the levels of G2/M related genes were analyzed. It was shown that α-tomatine decreased the expression of cyclin A and cyclin B1, while the p27 expression remained the same after treatment of α-tomatine (Figure 1F), reflecting the G2/M phase arrest after α-tomatine treatment in the way independent of p27. These data suggested that the proliferation, capacity of migration and invasion are significantly hindered by α-tomatine treatment in liver cancer HepG2 cells followed by G2/M phase arrest.

### α-tomatine reprograms transcriptomic networks through multi-pathway modulation in hepatocellular carcinoma

**FIGURE 2:**
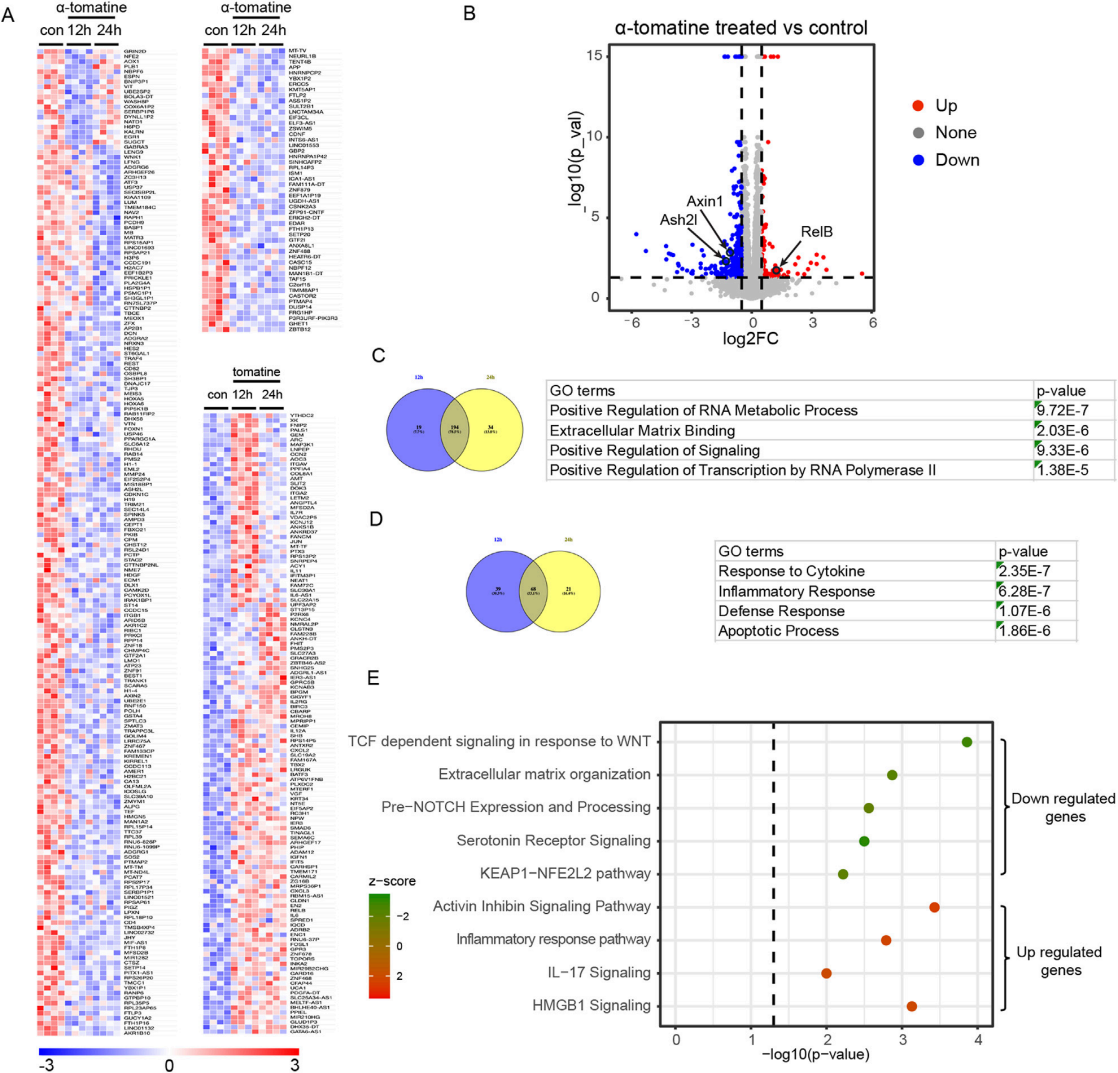
RNA-seq analysis of α-tomatine treatment in HepG2 cells. **(A)** Heatmap of the relative reduced or induced expression of 247 genes after 2 μM α-tomatine treatment for 12 h or 24 h in HepG2 cells. The expression was ≥1.5-fold over basal with adjusted p-value <0.05 and cut-off of FDR is 0.05. Blue to red indicate low to high expression levels, respectively. **(B)** Volcano plot of differential expressed genes after α-tomatine treatment in HepG2 cells. The expression was ≥1.5-fold over basal. Red spots indicate upregulated genes. Blue spots indicate downregulated genes. **(C)** 194 genes show reduced expression in both of 12h and 24 h after α-tomatine treatment in RNA-seq analysis of α-tomatine treatment in HepG2 cells. Gene ontology analysis identified the most enriched GO terms of all the genes with reduced expression after α-tomatine treatment. **(D)** 68 genes show induced expression in both of 12h and 24 h after α-tomatine treatment in HepG2 cells. Gene ontology analysis identified the most enriched GO terms of all the genes with induced expression after α-tomatine treatment. **(E)** IPA was conducted on either reduced or induced genes after α-tomatine treatment in HepG2 cells (P < 0.05, |log2FC| > 0.6). Four biological replicates for control, α-tomatine treatment for 12 h and α-tomatine treatment for 24 h.

To further study the outcomes and mechanisms of α-tomatine treatment in liver cancer cell, we performed RNA sequencing of HepG2 cell lines after treatment of α-tomatine for 12h and 24 h with four biological replicates in each group under 0.05 cut-off of False Discovery Rate (FDR). Heatmaps and volcano plot showed that α-tomatine treatment had a large effect on the expression of various genes, including 375 Differential Expressed Genes (DEGs) with fold change larger than 1.5 and adjusted p-value less than 0.05 (Figures 2A,B). Among 247 genes whose expression was reduced after α-tomatine treatment, the expression of 194 genes was attenuated in both 12h and 24 h of α-tomatine treatment (Figure 2C). The downregulated genes were associated with terms invoking RNA metabolic process, extracellular matrix binding, positive regulation of signaling and transcription by RNA polymerase II in gene ontology (GO) analysis (Figure 2C), which is consistent with the attenuated proliferation, migration and invasion in HepG2 cell lines after α-tomatine treatment. Moreover, α-tomatine resulted in increased expression of 128 genes, and the expression of 68 genes was elevated in both 12h and 24 h of α-tomatine treatment (Figure 2D). The GO analysis revealed the response to cytokine, inflammatory and defense response, apoptotic process among the top terms (Figure 2D). The Ingenuity Pathway Analysis (IPA) of differentially expressed genes further revealed a compromised activation of WNT response, extracellular matrix organization, NOTCH expression and processing, serotonin receptor signaling and KEAP1-NFE2L2 pathway in the downregulated genes, an enhanced activation of activin inhibin signaling pathway, inflammatory response pathway, IL17 signaling and HMGB1 signaling in the upregulated genes (Figure 2E). These data suggested that α-tomatine can induce or inhibit the expression of various genes which is involved in multiple processes related to proliferation, apoptosis and immune response.

### A-tomatine induces apoptosis and inhibits proliferation in HepG2 cells by attenuating wnt/β-catenin signaling

**FIGURE 3:**
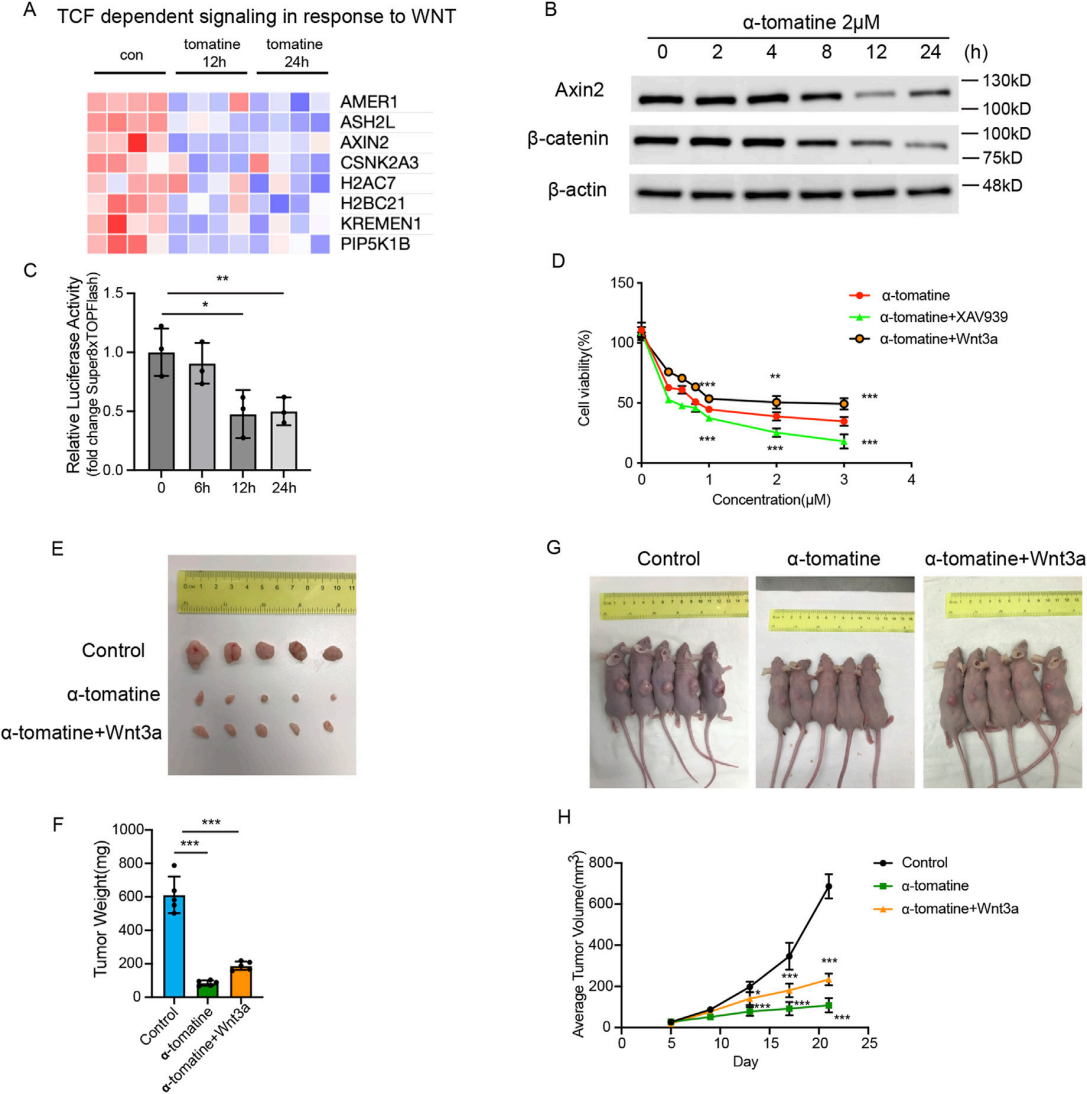
The Wnt/β-catenin signaling pathway limits the inhibitory effect of α-tomatine on HepG2 cells **(A)** Heatmap generated from the genes with reduced expression after α-tomatine treatment in RNA-seq linked to TCF dependent signaling in response to WNT. Blue to red indicate low to high expression levels, respectively. **(B)** The protein levels of Axin2 and β-catenin were determined by Western blot in HepG2 cells after treatment of 2 μM α-tomatine for different time points. **(C)** Super8XTOPFlash and Renilla luciferase plasmids were cotransfected into HepG2 cells and luciferase activity was measured before and after treatment of 2 μM α-tomatine for 6, 12 and 24 h. Three biological replicates. **(D)** Cell viability was measured by MTT assay in groups of 2 μM α-tomatine alone, α-tomatine and Wnt3a (250 ng/mL), α-tomatine and XAV939 (5 μM) for 24 h with different concentrations of α-tomatine. Three biological replicates. **(E)** Tumor tissues collected from randomly divided groups of BALB/c athymic nude mice inoculated with HepG2 cells were treated with vehicle solvent as control, α-tomatine (25 mg/kg) or α-tomatine (25 mg/kg) combined with Wnt3a (2 ug/kg) everyday by i.p. injection for 21 days. **(F)** Tumor weights were measured after collection of the tumors at 21 days **(G)** Tumor-bearing nude mice at 21 days inoculated with HepG2 cells under different treatment. **(H)** Tumor volumes were measured very 4 days. Tumor sizes, weights and volumes are expressed as the mean ± SEM (n = 5). Statistical significance was shown as *P < 0.05; **P < 0.01; ***P < 0.001.

Building upon RNA-seq findings demonstrating α-tomatine-induced impairment of WNT pathway activation (Figure 3A), we thought to characterize the functional involvement of canonical Wnt/β-catenin signaling in mediating α-tomatine’s therapeutic effects in HepG2 cells. Western blot quantification demonstrated that α-tomatine treatment significantly reduced Axin2 and β-catenin protein levels in a time-dependent manner (Figure 3B). Luciferase reporter assay by using Super8XTOPFlash reporter plasmid showed that WNT signaling activity reduced after α-tomatine treatment after 12 and 24 h in HepG2 cells (Figure 3C). Furthermore, activation of Wnt/β-catenin signaling by adding of Wnt3a significantly rescued cell viability induced by α-tomatine, whereas inhibition of Wnt/β-catenin signaling by adding of XAV939 markedly reduced cell viability (Figure 3D). To further validate the roles of Wnt signaling in the anti-tumor effects of α-tomatine, the HepG2 xenograft model was applied. The mice were randomized into Control group (s.c. injection of HepG2 cells; i.p. injection of corn oil; n = 5), α-tomatine group (s.c. injection of HepG2 cells; i.p. injection of α-tomatine; n = 5) and α-tomatine + Wnt3a group (s.c. injection of HepG2 cells; i.p. injection of α-tomatine combined with Wnt3a; n = 5). The *in vivo* experiment showed that the addition of Wnt3a during α-tomatine treatment result in larger tumor sizes and weights (Figures 3E–H). The above results demonstrated that the attenuated Wnt/β-catenin signaling is one of the mechanisms of α-tomatine that result in anti-proliferation in HepG2 cells.

### Upregulated RelB attenuates the inhibitory effect of α-tomatine on the proliferation of HepG2 cells

**FIGURE 4:**
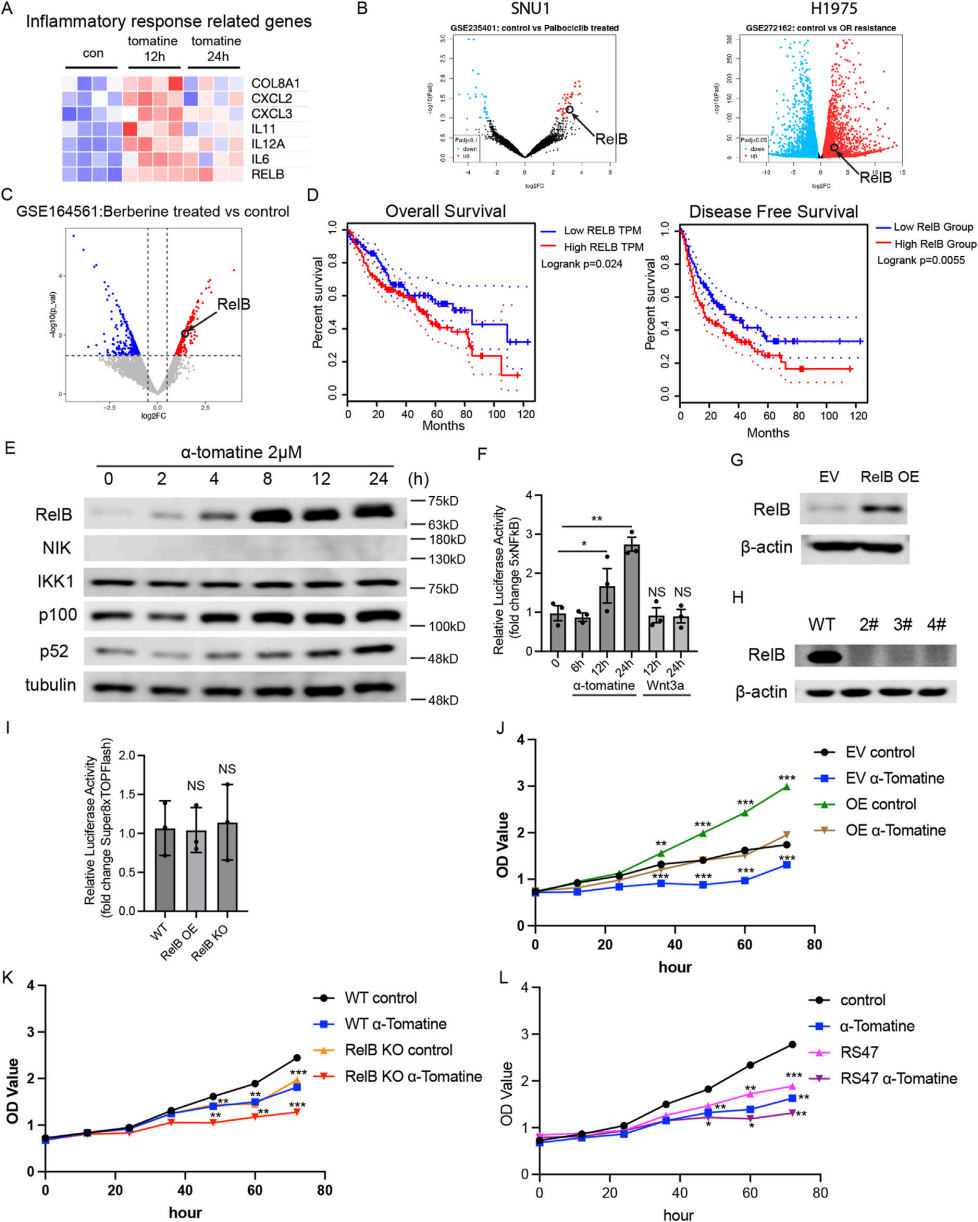
Upregulated RelB attenuates the inhibitory effect of α-tomatine on the proliferation of HepG2 cells **(A)** Heatmap generated from the genes with induced expression after α-tomatine treatment in RNA-seq linked to inflammatory response related genes. Blue to red indicate low to high expression levels, respectively. **(B)** Volcano plots of gene expression profile data in Gastric cancer cell SNU1 treated with Palbociclib in GSE235401 and Lung cancer cell H1975 showed resistance to Osimertinib in GSE272162 by GEO2R. **(C)** Volcano plots of gene expression profile data in liver cancer cell HepG2 treated with anti-tumor drug Berberine in GSE164561 by RStudio. **(D)** Kaplan-Meier analysis of overall survival and disease-free survival of liver hepatocellular carcinoma patients with low or high expression of RelB. Analysis was performed by Gene Expression Profiling Interactive Analysis (GEPIA2). n = 364. **(E)** The HepG2 cells were treated with 2 μM α-tomatine for different time point, and the protein levels of alternative NFkB pathway related proteins were measured by Western blot analysis. **(F)** 5XNFkB and Renilla luciferase plasmids were cotransfected into HepG2 cells and luciferase activity was measured before and after treatment of 2 μM α-tomatine for 6, 12 and 24 h or after treatment of 250 ng/mL Wnt3a for 12 and 24 h **(G)** RelB were overexpressed in HepG2 cells **(J)** the proliferation was measured by MTT kit after 2 μM α-tomatine treatment. Three biological replicates. **(H)** RelB were knocked out in HepG2 cells by CRISPR/Cas9 system, and **(K)** the proliferation was measured by MTT kit after 2 μM α-tomatine treatment. Three biological replicates. **(I)** Super8XTOPFlash and Renilla luciferase plasmids were cotransfected into WT, RelB overexpressed or RelB knockout HepG2 cells and luciferase activity was measured 16 h after cotransfection. **(L)** Proliferation of HepG2 cells was measured by MTT kit after treatment of 2 μM α-tomatine, 2 μM RelB inhibitor RS47 and combined treatment of 2 μM α-tomatine and RS47. Three biological replicates. All the values are shown as mean ± SEM. Statistical significance was shown as *P < 0.05; **P < 0.01; ***P < 0.001.

RNA-seq profiling revealed significant activation of cytokine storm-associated signaling in α-tomatine-treated HepG2 cells. Notably, the non-canonical NF-κB transcription factor RelB exhibited sustained upregulation at both 12h and 24 h post-treatment (Figure 4A). To explore if anti-tumor drugs also induce expression of RelB in other cancer cells, we searched GEO data sets. Upregulated DEGs in gastric cancer cell SNU1 treated with anti-tumor drug Palbociclib from GSE235401 and non-small cell lung cancer cell H1975 with anti-tumor drug Osimertinib resistance from GSE272162 revealed that RelB is one of the genes increased expression (Figure 4B). RelB was also shown in the upregulated DEGs in HepG2 cells treated with anti-tumor drug Berberine from GSE164561 (Figure 4C). Clinical correlation analysis demonstrated that hepatocellular carcinoma patients with high RelB expression exhibited significantly reduced overall survival and disease-free survival compared to low-expressing counterparts (Figure 4D). Western blot analysis confirmed α-tomatine-induced elevation of RelB and its upstream regulators NIK and phosphorylated p100, though p52 subunit expression remained unaltered (Figure 4E). Luciferase reporter assay by using 5xNFkB reporter plasmid showed that treatment of α-tomatine result in upregulation of NFkB luciferase activity, but Wnt3a stimulation did not alter NFkB luciferase activity in HepG2 cells (Figure 4F). To further elucidate the functional impact of RelB in the context of α-tomatine treatment, we genetically modulated its expression in HepG2 cells by either overexpressing or knocking it out using CRISPR/Cas9 technology (Figures 4G,H). Luciferase reporter assay by using Super8XTOPFlash reporter plasmid in RelB overexpressed or RelB knockout HepG2 cells did not show altered WNT signaling activity, indicating that α-tomatine might affect WNT and NFkB signaling pathways in independent manners. Overexpression of RelB led to increased proliferation of HepG2 cells in the absence of α-tomatine, while concurrently diminishing the antiproliferative efficacy of α-tomatine (Figure 4J). Conversely, RelB knockout in HepG2 cells curtailed their proliferative capacity without α-tomatine exposure and augmented the antiproliferative response to α-tomatine (Figure 4K). Interestingly, treatment of 2 μM RelB specific inhibitor RS47 (Li et al., 2025) alone lead to reduced proliferation of HepG2 cells, and combined treatment of RS47 and α-tomatine further augment the inhibitory effects of proliferation (Figure 4L). Collectively, these findings underscore a correlation between elevated RelB expression and poorer survival outcomes in liver hepatocellular carcinoma patients, highlighting that α-tomatine-induced RelB upregulation may compromise its therapeutic potential against HepG2 cell proliferation.

### RelB attenuates the anti-tumor efficiency of α-tomatine in HepG2 xenograft model

**FIGURE 5:**
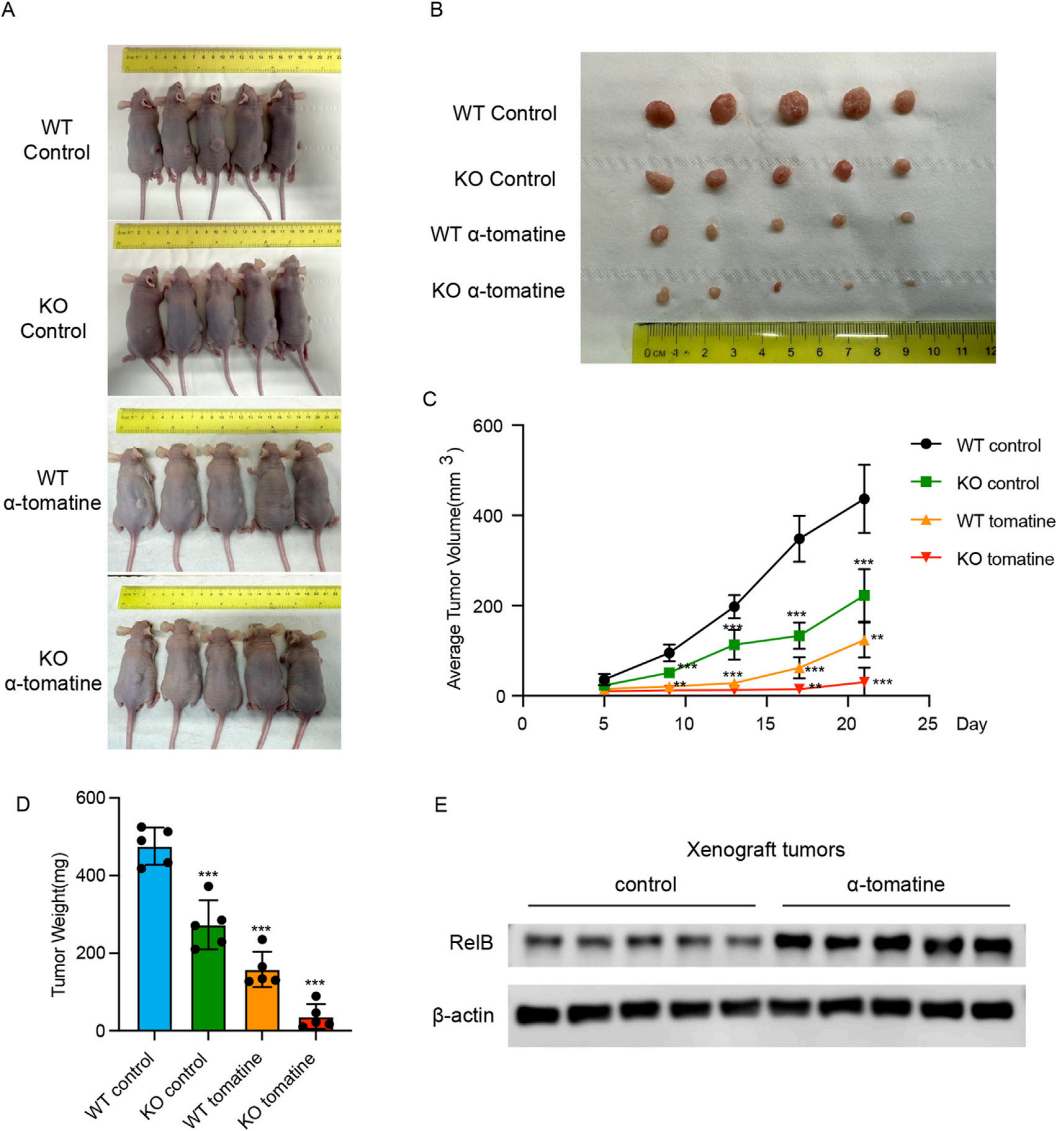
RelB attenuates the anti-tumor efficiency of α-tomatine in HepG2 xenograft model **(A)** Tumor-bearing nude mice and **(B)** tumor tissues collected from randomly divided BALB/c athymic nude mice groups inoculated with HepG2 WT or RelB KO cells were treated with vehicle solvent as control or α-tomatine (25 mg/kg) for 21 days n = 5 **(C)** Tumor volumes were measured very 4 days from 5 days to 21 days n = 5. **(D)** Tumor weights were measured after collection of the tumors at 21 days n = 5. **(E)** The expression of RelB was measured in the tumors of WT HepG2 cells after treatment with vehicle solvent or α-tomatine. Tumor sizes, weights and volumes are expressed as the mean ± SEM (n = 5).*P < 0.05; **P < 0.01; ***P < 0.001.

To further probe the impact of RelB on α-tomatine’s antitumor efficacy, we employed a HepG2 xenograft model for *in vivo* validation. Mice were stratified into five cohorts: WT control (s.c. injected with WT HepG2 cells; i.p. administered corn oil; n = 5), KO control (s.c. injected with RelB KO HepG2 cells; i.p. administered corn oil; n = 5), WT tomatine (s.c. injected with WT HepG2 cells; i.p. treated with α-tomatine; n = 5), and KO tomatine (s.c. injected with RelB KO HepG2 cells; i.p. treated with α-tomatine; n = 5). Treatments—corn oil as vehicle or α-tomatine—were administered daily for 21 consecutive days. The data revealed that while α-tomatine treatment alone or RelB deficiency suppressed tumor volume in WT HepG2 models, combining α-tomatine with RelB KO HepG2 cells led to a significant reduction in tumor volume (Figures 5A–C). Additionally, α-tomatine treatment markedly decreased tumor weights in RelB KO HepG2 mice (Figure 5D). Notably, RelB expression within tumors was upregulated following 21 days of α-tomatine exposure (Figure 5E). In summary, our findings illustrate that α-tomatine-induced elevation of RelB diminishes its *in vivo* antitumor effectiveness.

## Discussion

α-Tomatine, within a concentration range of 0.1–100 μg/mL, has emerged as a potent dose-dependent inhibitor, suppressing the proliferation of liver cancer HepG2 cell lines by 46.3%–89.2%. Notably, its anticarcinogenic potency at 1 μg/mL surpasses that of doxorubicin in human liver cancer cells (Lee et al., 2004). Furthermore, α-tomatine has been documented to impede the growth of various human cancer cell lines (Friedman et al., 2009), induce apoptosis in androgen-independent prostate adenocarcinoma PC-3 cells (Lee et al., 2011) and provoke growth arrest and apoptosis in leukemia HL-60 cells (Huang et al., 2015a). Mechanistic insights indicate that α-tomatine’s inhibition of HCC tumorigenesis is partially mediated through the modulation of p53, calcium efflux pumps, expression of anti-apoptotic proteins and reactive oxygen species (ROS) signaling pathways in response to cellular stress (Echeverria et al., 2022). The α-tomatine might induce paradoxically resistance signaling networks in addition to its inhibition of tumor growth, the effects may differ in early and late-stages of HCC, or in different microenvironments such as hypoxia and inflammatory conditions (Zheng et al., 2025). It could be very difficult to decipher the primary and compensatory signaling pathways mediated by α-tomatine since many pathways like Nrf2, MAPK, VEGFR and PI3K/AKT have dual roles of providing protection in normal liver cells but supporting survival of HCC during chemotherapy (Gan et al., 2024; Xue et al., 2024). Therefore, the comprehensive mechanisms underlying its antitumor action on liver cancer cells necessitate further elucidation.

Emerging evidence posits that apoptosis induction and DNA damage may be mediated via Wnt/β-catenin signaling pathway inhibition (Fu et al., 2017), while its aberrant activation can counteract anti-tumor drug-induced apoptosis (Liang et al., 2017; Lin et al., 2016). Nonetheless, the impact of α-tomatine on this pathway remained unclear. Our findings reveal that α-tomatine attenuates HepG2 cells’ responsiveness to WNT stimuli and dampens Wnt/β-catenin pathway activity in luciferase reporter assay. Notably, inhibiting this pathway bolsters α-tomatine’s anti-tumor efficacy, whereas Wnt3a supplementation impedes it. The antiproliferative and cytotoxic effects of α-tomatine are partly attributable to its Wnt/β-catenin pathway downregulation. However, α-tomatine does not completely stymie the pathway, as further inhibition by XAV939 still curbs tumor growth. Additional investigations are warranted to elucidate α-tomatine’s role in modulating Wnt responses comprehensively. Moreover, the specific contributions of altered RNA metabolism, extracellular matrix organization, Notch signaling, serotonin receptor signaling, and Keap1-Nfe2l2 pathway, as unveiled by our RNA-seq analysis, to α-tomatine’s anti-tumor efficacy necessitate further exploration.

NF-κB transcription factors are major players in the control of cell proliferation and survival of numerous cancers (Baldwin, 2012; Baud and Karin, 2009; Ben-Neriah and Karin, 2011; Eluard et al., 2020; Nakanishi and Toi, 2005; Naugler and Karin, 2008). Although NF-κB activation is a frequent and early event in human liver cancers of viral or nonviral etiologies and has been associated with the acquisition of a transformed phenotype during hepatocarcinogenesis (Liu et al., 2002), but the roles of RelB in the efficiency of anti-tumor drugs in liver cancers have never been studied. Our study clearly shows that the expression of RelB is induced after treatment of α-tomatine in HepG2 cells *in vitro* and *in vivo*. And α-tomatine treatment upregulates NFkB activity in luciferase reporter assay. Clinical evidence further correlates high RelB expression with diminished overall survival rates in liver hepatocellular carcinoma patients. Within HepG2 cells, RelB overexpression mitigates α-tomatine’s anti-tumor effects, whereas RelB depletion amplifies these effects. Crucially, the *in vivo* growth-suppressive impact of α-tomatine on HepG2 tumors is augmented by RelB depletion. While it's acknowledged that RelB downregulation suppresses HepG2 cell proliferation—a phenomenon observed in numerous other cancer forms (Jacque et al., 2013; Shen et al., 2018; Zhou et al., 2018), our findings introduce novel insights: elevated RelB expression reduced the anti-tumor activity of α-tomatine in HepG2 cells. Precedent studies have implicated RelB in fostering resistance to TAM and DOXO in breast cancer (Mineva et al., 2009; Xu et al., 2023), suggesting that RelB-mediated drug resistance could potentially elucidate the poorer prognosis observed in liver cancer patients exhibiting high RelB levels. Our data further revealed that combination of RelB inhibitor RS47 and α-tomatine further augment the inhibitory roles of α-tomatine, suggesting potential clinical relevance in HCC therapy. Nevertheless, comprehensive experimental investigations are imperative to ascertain the broader implications of RelB in mediating resistance to various anti-tumor drugs across a spectrum of liver cancer cell lines. Interestingly, the Wnt/β-catenin signaling pathway and RelB dependent NFkB signaling pathway does not affect each other in HepG2 cells directly, revealing those two mechanisms act independently.

However, there are still some potential limitations in this study. Our HepG2 xenograft model can not reflect the roles of realistic tumor microenvironments or liver-specific pharmacokinetics. Moreover, HepG2 cell does not represent other subtypes of liver cancer, therefore we do not know if α-tomatine exerts anti-tumor activity in other liver cancer subtypes. It is important to note that the test of toxicology is undergoing transformation with advanced in multiple testing systems and high-throughput assays that enable the prediction of toxicological responses to guide their safe translation into therapeutic applications with the purpose to reduce variations of batch effects, assay conditions or measurement errors (Dhage et al., 2021; Mone et al., 2023; Singh et al., 2024; Tiwari Pandey et al., 2021). Further study will be required to determine the outcomes of α-tomatine on normal cells and tissues for the test of inflammatory responses and toxic risk assessment before clinical applications (Chandrasekar et al., 2025; Chandrasekar et al., 2024; Chandrasekar et al., 2022; Singh et al., 2023).

**FIGURE 6:**
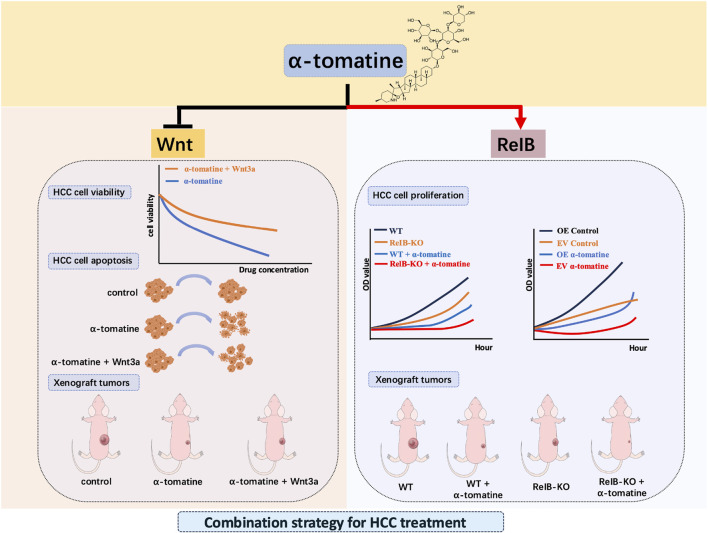
Graphical abstract for the dual-edged mechanisms of α-tomatine in hepatocellular carcinoma.

In summary, we explored the potential anti-tumor mechanisms of α-tomatine in HepG2 cell lines and observed that the downregulation of the Wnt/β-catenin signaling pathway may contribute to its anti-tumor effects. Additionally, our findings suggest that increased expression of RelB induced by α-tomatine might be associated with reduced sensitivity to its anti-tumor activity. These results indicate a complex, dual role of α-tomatine in liver cancer (Figure 6). Combination of α-tomatine and curcumin or paclitaxel more potently inhibited the growth of PC-3 tumors than either agent alone (Huang et al., 2015b; Lee et al., 2013). These findings highlight the possibility of developing novel cancer treatment strategies by leveraging the synergistic anti-tumor effects of α-tomatine with other natural compounds or chemotherapy drugs. Our data provide preliminary insights into the functional role of α-tomatine in liver cancer and offer a potential rationale for further investigation into targeting RelB in combination therapies for liver cancer treatment.

## Conclusion

This study elucidates the dual mechanisms of α-tomatine in hepatocellular carcinoma (HCC), demonstrating its potent anti-tumor effects through suppression of the Wnt/β-catenin signaling pathway while uncovering RelB-mediated resistance as a limiting factor in its therapeutic efficacy in independent mechanisms. α-tomatine suppresses Wnt/β-catenin signaling via β-catenin phosphorylation and degradation, contributing to its anti-cancer activity. Notably, genetic ablation of RelB enhanced α-tomatine’s anti-tumor effects both *in vitro* and *in vivo*, whereas RelB overexpression conferred resistance, underscoring its role as a critical mediator of therapeutic escape. Our study provides a novel framework for combining α-tomatine with RelB inhibition as a synergistic phytotherapy approach in HCC. Future research should explore the clinical potential of this dual pathway targeting strategy, particularly in overcoming resistance mechanisms in liver cancer treatment. By integrating botanical pharmacodynamics with precision oncology, this work advances the development of more effective, resistance-evading therapeutic regimens for HCC.

## Data Availability

The original contributions presented in the study are publicly available. This data can be found here: GEO repository, under the accession number GSE308098.

## Abbreviations

CCA, cholangiocarcinoma; ERK, extracellular signal-regulated kinase; EZH2, enhancer of zeste homology 2; GO, gene ontology; HCC, hepatocellular carcinoma; IPA, ingenuity pathway analysis; TACE, transarterial chemoembolization; PI3K/Akt, phosphoinositide 3-kinase/protein kinase B; NSCLC, non-small cell lung cancer; ROS, reactive oxygen species; KO, knock out; OE, overexpression.
